# Meta-Review of CSF Core Biomarkers in Alzheimer’s Disease: The State-of-the-Art after the New Revised Diagnostic Criteria

**DOI:** 10.3389/fnagi.2014.00047

**Published:** 2014-03-24

**Authors:** Daniel Ferreira, Lilisbeth Perestelo-Pérez, Eric Westman, Lars-Olof Wahlund, Antonio Sarría, Pedro Serrano-Aguilar

**Affiliations:** ^1^Section of Clinical Geriatrics, Department of Neurobiology, Care Sciences and Society, Karolinska Institutet, Stockholm, Sweden; ^2^Evaluation Unit of the Canary Islands Health Service, Santa Cruz de Tenerife, Spain; ^3^Red de Investigación en Servicios de Salud en Enfermedades Crónicas, Santa Cruz de Tenerife, Spain; ^4^Agency for Health Technology Assessment, Institute of Health Carlos III, Madrid, Spain

**Keywords:** Alzheimer’s disease, cerebrospinal fluid biomarkers, amyloid beta-protein (42), tau protein, sensitivity, specificity, meta-review, state-of-the-art review

## Abstract

**Background:** Current research criteria for Alzheimer’s disease (AD) include cerebrospinal fluid (CSF) biomarkers into the diagnostic algorithm. However, spreading their use to the clinical routine is still questionable.

**Objective:** To provide an updated, systematic and critical review on the diagnostic utility of the CSF core biomarkers for AD.

**Data sources:** MEDLINE, PreMedline, EMBASE, PsycInfo, CINAHL, Cochrane Library, and CRD.

**Eligibility criteria:** (1a) Systematic reviews with meta-analysis; (1b) Primary studies published after the new revised diagnostic criteria; (2) Evaluation of the diagnostic performance of at least one CSF core biomarker.

**Results:** The diagnostic performance of CSF biomarkers is generally satisfactory. They are optimal for discriminating AD patients from healthy controls. Their combination may also be suitable for mild cognitive impairment (MCI) prognosis. However, CSF biomarkers fail to distinguish AD from other forms of dementia.

**Limitations:** (1) Use of clinical diagnosis as standard instead of pathological postmortem confirmation; (2) variability of methodological aspects; (3) insufficiently long follow-up periods in MCI studies; and (4) lower diagnostic accuracy in primary care compared with memory clinics.

**Conclusion:** Additional work needs to be done to validate the application of CSF core biomarkers as they are proposed in the new revised diagnostic criteria. The use of CSF core biomarkers in clinical routine is more likely if these limitations are overcome. Early diagnosis is going to be of utmost importance when effective pharmacological treatment will be available and the CSF core biomarkers can also be implemented in clinical trials for drug development.

## 1 Introduction

Dementia is becoming a worldwide problem causing a tremendous burden to the public health system and society (http://www.alz.org/documents_custom/trajectory.pdf; Ferri et al., 2010). Among different types of dementia, Alzheimer’s disease (AD) is the most common form, affecting more than 27 million people and accounting for 60–70% of all dementia cases (Hebert et al., 2003; Brookmeyer et al., 2007). Therefore, effective strategies for early diagnosis, prevention and treatment are urgently needed. Regarding diagnosis, the clinical criteria established in 1984 by the NINCDS–ADRDA (McKhann et al., 1984) has recently been revised by the National Institute on Aging and the Alzheimer Association (Jack et al., 2011; McKhann et al., 2011). These criteria for AD incorporate two notable differences. First, the AD process is considered as a continuum that encompasses three different disease stages: (1) preclinical phase, in which subjects are cognitively normal but have AD pathology; (2) symptomatic pre-dementia phase: mild cognitive impairment (MCI); and (3) dementia phase: AD (Jack et al., 2011). Second, this pathophysiological process can be studied *in vivo* by means of different biomarkers.

A biomarker is a measurable biological feature that can be used to diagnose or predict a physiological or pathological condition (Barber, 2010). Main AD biomarkers investigated so far may be broken into two classes based on the biological aspect they measure. Biomarkers of brain amyloid-beta (Aβ) protein depositions are low cerebrospinal fluid (CSF) Aβ_42_ and positive PET amyloid imaging (Jack et al., 2008; Chételat et al., 2010). Biomarkers of downstream neuronal degeneration or injury are elevated CSF tau (both total tau and hyperphosphorylated tau: p-tau); decreased 18 fluorodeoxyglucose (FDG) uptake on PET in temporo–parietal cortex; and disproportionate atrophy on structural magnetic resonance imaging (MRI) in medial, basal, and lateral temporal lobe, and medial parietal cortex. These biomarkers have been integrated into a hypothetical model published by Jack et al. (2010a). According to this model, biomarkers of Aβ accumulation become abnormal first, being Aβ accumulation necessary but not sufficient to produce the clinical symptoms of MCI and dementia. Biomarkers of neuronal injury and neurodegeneration are abnormal later, retaining a close relationship with cognitive performance through the clinical phases of MCI and dementia (Vemuri et al., 2010). However, autopsy data suggest that tau pathophysiology might precede Aβ deposition (Braak and Del Tredici, 2011). This apparently conflicting evidence has been integrated in a recent revision of the model (Jack et al., 2013). Aβ and tau pathophysiological processes might be initiated independently in sporadic AD. Subcortical tauopathy might occur first although it is only detectable by immunostaining methods. Aβ pathophysiology arises later and independently from pre-existing tauopathy. Through unknown mechanisms, Aβ pathophysiology would accelerate the antecedent subcortical tauopathy leading to neocortical spread of neurofibrillary tangles (Jack et al., 2013).

This meta-review is focused on CSF biomarkers. Although significant advances have been made in the field of neuroimaging, biomarkers based on CSF are at present the most convenient for studying disease progression (Hampel et al., 2008; Anoop et al., 2010; Monge-Argilés et al., 2010). CSF biomarkers reflect key neuropathological hallmarks of AD, i.e., amyloid plaques and neurofibrillary tangles (Braak and Braak, 1991; Thal et al., 2002). Accumulation of amyloid plaques and neurofibrillary tangles probably starts 20–30 years before the clinical onset of the disease. Therefore, CSF biomarkers are the most suitable candidates to facilitate AD diagnosis in the very early stages of the disease, long before symptoms onset. Moreover, since it may be optimal to treat the neuropathology as early as possible, biomarkers of preclinical AD are likely to play a pivotal role in the development of the next generation of therapies.

Numerous studies on CSF biomarkers for AD have been published during the last years, however frequently providing contradictory and inconclusive results. In this sense, the fact of spreading the use of CSF biomarkers to the clinical routine is still questionable. An effort has not been done yet to systematically define the state-of-the-art since the new revised research criteria for AD were published in May 2011. It is therefore timely and highly necessary to integrate all the information available in the literature, evaluate the findings, and assess the diagnostic efficiency of CSF biomarkers. Only in this sense it will be possible to answer the relevant question of for which patients these CSF biomarkers can be useful in the clinical practice.

## 2 Objectives

Since the CSF core biomarkers have been incorporated to the current diagnostic criteria for AD for complementing clinical impression with biological support of AD pathology, the primary objective of this meta-review is to present an updated systematic and critical review on the diagnostic performance of the CSF core biomarkers for AD (Aβ_42_, T-tau, and p-tau).

In particular, we aim to answer three specific questions. The first two addresses the issue of AD diagnosis and the third one is related to AD prediction:

(1) What is the diagnostic efficiency of CSF Aβ_42_, T-tau, and p-tau for the diagnosis of AD vs. healthy controls?
(2) What is the diagnostic efficiency of CSF Aβ_42_, T-tau, and p-tau for the diagnosis of AD vs. other dementias: dementia with Lewy bodies (DLB), frontotemporal lobar degeneration (FTLD), vascular dementia (VaD), and Creutzfeldt–Jakob disease (CJD)?
(3) What is the diagnostic efficiency of CSF Aβ_42_, T-tau, and p-tau for the early detection of MCI patients that will progress to AD vs. MCI patients that will remain stable over time?

In order to address these questions, we reviewed systematic reviews with meta-analysis as well as primary studies published after the publication of the new revised diagnostic criteria. These studies include case–control studies with prospective or retrospective, cross-sectional or longitudinal designs.

## 3 Materials and Methods

### 3.1 Search methods

A systematic review was conducted for the period between January 1990 and September 2013. Consulted electronic databases were MEDLINE and PreMedline, EMBASE, PsycInfo, CINAHL, Cochrane Library, and CRD. The search strategy was developed for each database using the combination of the following medical subject heading (MeSH) and free-text terms: “AD diagnosis” or “AD”, and “abeta-42” or “T-tau” or “P-tau” or “tau” or “phospho-tau” or “phosphorylated tau”. Examples of the search strategy followed for the two major databases are shown in Table A1 in Appendix (MEDLINE–OVID) and Table A2 in Appendix (EMBASE–Elsevier). In addition, reference sections of included reports were searched to identify relevant publications. Researchers thought likely to have carried out relevant studies were also contacted. Studies addressing CSF Aβ_42_, T-tau, and p-tau in AD but primarily focusing in other conditions were also covered.

### 3.2 Study selection

Initial inclusion criteria for the current review were studies that: (1) included a systematic review with meta-analysis; (2) evaluated the diagnostic performance of at least one of the CSF core biomarkers for AD (Aβ_42_, T-tau, and/or P-tau); and (3) were published in English or Spanish. Exclusion criteria were studies that: (1) did not follow a rigorous process of systematic review (defining the question, finding the evidence, documenting the search process, and appraising and selecting suitable studies), and (2) did not provide any meta-analysis.

**Figure 1:**
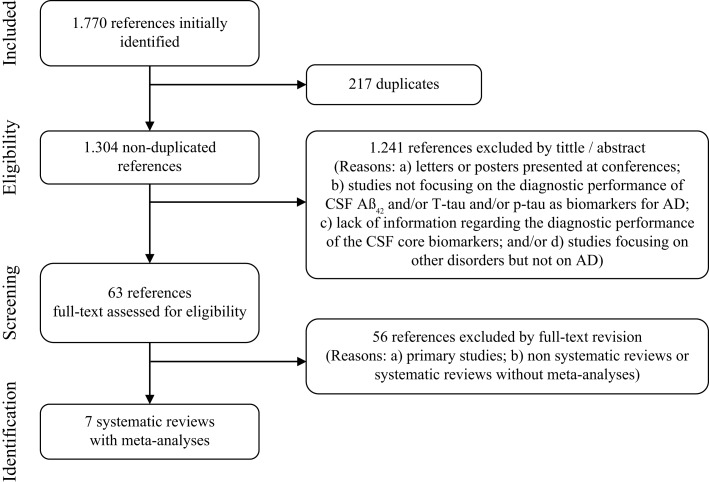
**Study selection flow for systematic reviews with meta-analyses**.

Two reviewers performed the study selection (Daniel Ferreira, Lilisbeth Perestelo-Perez). Peer review was done independently. In case of doubt and/or disagreements a third reviewer was consulted (Pedro Serrano-Aguilar). A total of 1,770 records were identified in the initial search. Duplicated articles were removed and remaining 1,304 publications were screened from title and abstract according to selection criteria. Sixty-three potentially relevant studies were then gathered and full text examined. Finally, seven articles completely fulfilled the selection criteria: Bloudek et al. (2011), Diniz et al. (2008), Mitchell (2009), Monge-Argilés et al. (2010), Schmand et al. (2010); Sunderland et al. (2003), and Van Harten et al. (2011). They all were systematic reviews with meta-analyses. Selection flow including reasons for study exclusion at each phase is fully detailed in Figure 1.

**Figure 2:**
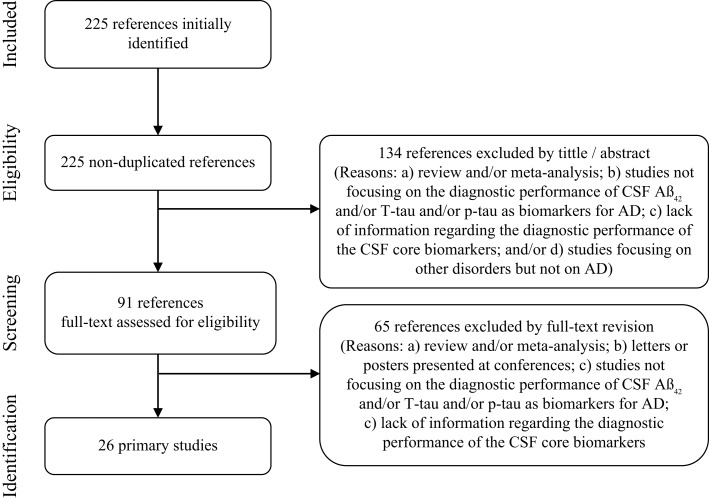
**Study selection flow for primary studies published after the new revised diagnostic criteria**.

As noted above, the original scope of this meta-review was to identify systematic reviews with meta-analyses. However, we did not detect any of these studies published after the new revised criteria for AD (May 2011). Hence, in order to synthesize the available evidence from May 2011 to the date of our search (September 2013), we decided to carry out a specific search for primary studies. Inclusion criteria were studies that: (1) were accepted and/or published after May 2011; (2) evaluated the diagnostic performance of at least one of the CSF core biomarkers for AD (Aβ_42_, T-tau, and/or P-tau); and (3) were published in English or Spanish. Same combination of MeSH and free-text terms was applied although including specifications for primary studies. From a total of 220 records, 26 studies fulfilled inclusion criteria and were selected for this specific evidence-based synthesis. Complete selection flow and reasons for study exclusion are fully detailed in Figure 2.

### 3.3 Data collection, risk of bias and evaluation of methodological quality

A data extraction sheet was developed to collect relevant data by covering: author and publication year, country, objectives, search methods, study selection, study design, CSF biomarkers evaluated, characteristics of diagnostic groups, statistical analyses, results (diagnostic accuracy and main findings), and conclusions. Data extraction was carried out by a single researcher (Daniel Ferreira) for each eligible study. A second researcher verified the extracted data with the original sources to ensure the quality and accuracy of the extraction (Lilisbeth Perestelo-Pérez).

Differential handling of positive results compared to negative results lead to a misleading bias in the overall published literature. Therefore, published studies may not be truly representative of all valid studies undertaken, and this bias may affect systematic reviews and meta-analyses. Several strategies were thus followed in order to reduce the risk of bias related to publication, data availability, and reviewer selection (see Table A3 in Appendix). Moreover, users’ guides published by Oxman et al. (1994) and PRISMA statement (Liberati et al., 2009; Moher et al., 2009) were used to critically evaluate the methodological quality of included systematic reviews with meta-analyses. Finally, this study was performed in accordance with the PRISMA statement, which provides a detailed guideline of preferred reporting style for systematic reviews and meta-analyses.

### 3.4 Outcome measures and statistical analyses

For each CSF core biomarker (Aβ_42_, T-tau, and p-tau), the following outcome measures of diagnostic ability were considered: sensitivity, specificity, and diagnostic accuracy [positive predictive value (PPV); negative predictive value (NPV)]. Means, standard deviations, and maximum and minimum values for sensitivity and specificity were calculated for primary studies. In addition, likelihood ratios were calculated from mean sensitivity and specificity values provided in the systematic reviews with meta-analysis and primary studies:

$$\begin{array}{c} \text{Positive likelihood ratio }(\text{LR}+)=\text{sensitivity}/(1-\text{specificity})\\ \text{Negative likelihood ratio }(\text{LR}-)=(1-\text{sensitivity})/\text{specificity} \end{array}$$

## 4 Results

### 4.1 Systematic reviews with meta-analysis

#### 4.1.1 Main characteristics of studies and methodological quality

Among the seven systematic reviews with meta-analyses included in this meta-review, three refer to the diagnostic ability of CSF core biomarkers to discriminate AD vs. healthy controls (Sunderland et al., 2003; Mitchell, 2009; Bloudek et al., 2011). Three studies include patients with AD vs. other dementias (Mitchell, 2009; Bloudek et al., 2011; Van Harten et al., 2011). More specifically, Bloudek et al. (2011) and Mitchell (2009), compared AD to a mixed group including different types of dementia. Only Van Harten et al. (2011) specified separated groups of dementia: DLB, FTLD, VaD, and CJD. Finally, four studies describe the ability of CSF core biomarkers to differentiate patients with MCI who progress to AD or other dementias [MCI-converters (MCI-C)] compared with patients with MCI who remain stable over time [MCI-stable (MCI-S)] (Diniz et al., 2008; Mitchell, 2009; Monge-Argilés et al., 2010; Schmand et al., 2010). Table 1 summarizes the main characteristics of each selected systematic review with meta-analysis.

**Table 1:** **Main characteristics of selected systematic reviews with meta-analyses**.

Reference (country)	Consulted databases (period)	Criteria for study selection	Biomarkers	Diagnostic groups compared	Number of studies included in the meta-analyses
Bloudek et al. (2011) (USA)	MEDLINE (January 1990–March 2010)	Not specified. Different thresholds for CSF biomarkers interpretation are combined. p-tau epitopes are also combined (181, 199, 231)	Aβ_42_	AD vs. controls	AD vs. controls
			T-tau	AD vs. other dementias	Aβ_42_: 14
			p-tau		T-tau: 22
			Combination		p-tau: 14
					Combination: 11
					AD vs. other dementias
					Aβ_42_: 11
					T-tau: 15
					p-tau: 14
					Combination: 7
Diniz et al. (2008) (Brazil)	MEDLINE, EMBASE, and PsycINFO (January 1999–April 2007)	(1) MCI criteria according Petersen et al. (1999), or compatible; (2) information about conversion to dementia (from controls or MCI); (3) information regarding the follow-up period; (4) baseline levels of T-tau and/or p-tau and/or Aβ_42_ for MCI that will progress to AD; (5) when two studies had overlapping samples, the one with biggest sample was chosen	Aβ_42_ T-tau p-tau	MCI-C vs. MCI-S^a^ Aβ_42_: MCI-C (*n* = 130), MCI-S (*n* = 190) T-tau: MCI-C (*n* = 149), MCI-S (*n* = 205) p-tau: MCI-C (*n* = 101), MCI-S (*n* = 167)	MCI-C vs. MCI-S Aβ_42_: 4 T-tau: 5 p-tau: 3
Mitchell (2009) (UK)	MEDLINE, PsycINFO, ASSIA, EMBASE, Science Direct, Ingenta Select, Ovid, Web of Knowledge, and Wiley–Blackwell (until February 2009)	(1) All studies were evaluated and filtered by STARD criteria and guidelines for diagnostic testing of evidence-based medicine; (2) AD criteria according NINCDS–ADRDA; (3) MCI criteria according Mayo Clinic (i.e., Petersen), or prospective validation after 1 year follow-up); (4) p-tau epitopes are combined (181, 199, 231); (5) studies with data previously published in other works are not included in the MA	p-tau	AD (*n* = 329) vs. controls^b^ (*n* = 971) MCI-C (*n* = 163) vs. MCI-S (*n* = 225) AD (*n* = 1304) vs. other dementias (*n* = 488)	AD vs. controls = 19 MCI-C vs. MCI-S: 6 EA vs. other dementias: 18
Monge-Argilés et al. (2010) (Spain)	PubMed and EMBASE (January 1999–September 2008)	(1) MCI criteria according Petersen et al. (1999), or any other clearly defined criteria; (2) AD criteria according NINCDS–ADRDA and DSM-IV; (3) information regarding the follow-up period for MCI patients; (4) baseline levels of T-tau and/or p-tau and/or Aβ_42_ for MCI that will progress to AD; (5) sensitivity and specificity values	Aβ_42_	MCI-C vs. MCI-S	MCI-C vs. MCI-S
			T-tau	Aβ_42_: MCI-C (*n* = 128), MCI-S (*n* = 216)	Aβ_42_: 6
			p-tau	T-tau: MCI-C (*n* = 213), MCI-S (*n* = 383)	T-tau: 11
			Combination	p-tau: MCI-C (*n* = 157), MCI-S (*n* = 306)	p-tau: 7
				Combination: MCI-C (*n* = 101), MCI-S (*n* = 164)	Combination: 3
Schmand et al. (2010) (Netherlands)	PubMed, MEDLINE, EMBASE, and PsycINFO (January 2003–November 2008)	(1) Longitudinal designs; (2) healthy controls or MCI at baseline; (3) conversion to MCI and/or AD at follow-up; (4) MCI criteria according Petersen et al. (1999, 2001); (5) AD criteria according McKhann et al. (1984) or CDR; (5) baseline levels of CSF biomarkers; (6) studies combining several biomarkers were excluded if information for separated biomarkers was not provided; (7) when two studies had overlapping samples, the one with biggest sample was chosen	Aβ_42_ T-tau p-tau	MCI-C^c^ Aβ_42_: *n* = 514 T-tau: *n* = 697 p-tau: *n* = 616	MCI-C Aβ_42_: 9 T-tau: 12 p-tau: 10
Sunderland et al. (2003) (USA)	PubMed y MEDLINE (August 1989–March 2003)	Exclusion criteria: (1) Lack of information about controls; (2) diagnostic criteria not specified or mixed criteria; (3) different Aβ components not distinguished (40–42); (4) studies with *n* < 25 participants or standard deviations for Aβ not provided, (5) when two studies had overlapping samples, the most complete one was chosen	Aβ_42_ T-tau	AD vs. controls Aβ_42_: AD (*n* = 849), controls (*n* = 427) T-tau: AD (*n* = 2284), controls (*n* = 1054)	AD vs. controls Aβ_42_: 17 T-tau: 34
Van Harten et al. (2011) (Netherlands)	PubMed (until July 2010)	Articles that: (1) are addressed to improve the diagnosis of dementia by means of CSF biomarkers; (2) include patients afflicted with DLB, FTLD, VaD, or CJD. Mixed groups of dementia are excluded; (3) include measures of T-tau and/or p-tau; (4) perform meta-analyses of at least four studies; (5) have more than three patients per diagnostic group; (6) when two studies had overlapping samples, the one with biggest sample was chosen; (7) regarding CJD, studies were included when majority of patients were sporadic CJD	T-tau	AD vs. DLB	AD vs. DLB
			p-tau	T-tau: AD (*n* = 473), DLB (*n* = 208)	T-tau: 19
				p-tau: AD (*n* = 531), DLB (*n* = 210)	p-tau: 9
				AD vs. FTLD	AD vs. FTLD
				T-tau: AD (*n* = 1330), FTLD (*n* = 464)	T-tau: 22
				p-tau: AD (*n* = 607), FTLD (*n* = 249)	p-tau: 7
				AD vs. VaD	AD vs. VaD
				T-tau: AD (*n* = 1048), VaD (*n* = 306)	T-tau: 23
				p-tau: AD (*n* = 333), VaD (*n* = 165)	p-tau: 8
				AD vs. CJD	AD vs. CJD
				T-tau: AD (*n* = 175), CJD (*n* = 110)	T-tau: 7

*AD, Alzheimer’s disease; MCI, mild cognitive impairment (MCI-C: MCI-converters, MCI-S: MCI-stables); DLB, dementia with Lewy bodies; FTLD, frontotemporal lobe degeneration; VaD, vascular dementia; CJD, Creutzfeldt–Jakob disease*.

*^a^Some studies include healthy controls together with MCI-S patients*.

*^b^Controls (participants without cognitive impairment but may have significant neurological disease)*.

*^c^Some studies include healthy controls together with MCI patients at baseline. At follow-up, some studies analyze MCI who progress to any type of dementia, not necessarily AD*.

Scores in the Oxman’s scale are presented in Table A4 in Appendix. All the included systematic reviews with meta-analysis had total scores between 7 and 8, which correspond to satisfactory methodological quality (Oxman et al., 1994). PRISMA checklist is presented in Table A5 in Appendix, showing reporting transparence of the different systematic reviews.

#### 4.1.2 Diagnostic performance of CSF core biomarkers for AD

Results from included systematic reviews with meta-analysis are presented below according to the specific objectives of this meta-review. The first section includes the comparison between AD and healthy controls. The second section details the differential diagnosis between AD and other dementias. The third and last section addresses the discrimination between MCI-C and MCI-S. For each section, information is presented separately for the three different biomarkers as well as possible combinations between them. Tables 2–4 summarize mean sensitivity and specificity values provided in the various meta-analyses, as well as corresponding likelihood ratios.

**Table 2:** **Systematic reviews with meta-analyses (AD vs. healthy controls): sensitivity and specificity values**.

CSF biomarker and study	Number of studies included	Number of AD patients	Number of healthy controls	Sensitivity (95% CI)	Specificity (95% CI)	LR+	LR−
**Aβ_42_**							
Bloudek et al. (2011)	14	ns	ns	80 (73–85)	82 (74–88)	4	0.2
**T-TAU**							
Bloudek et al. (2011)	22	ns	ns	82 (76–87)	90 (86–93)	8	0.2
**p-TAU**							
Mitchell (2009)	19	1329	971	78 (71–84)	88 (84–91)	7	0.3
Bloudek et al. (2011)	14	ns	ns	80 (70–87)	83 (75–88)	5	0.2
**COMBINATION OF Aβ_42_ AND T-TAU**							
Bloudek et al. (2011)	11	ns	ns	89 (84–92)	87 (83–90)	7	0.1

*AD, Alzheimer’s disease; CI, confidence interval; LR+, positive likelihood ratio; LR−, negative likelihood ratio; ns, non-specified; sensitivity and specificity values are expressed in percentages*.

**Table 3:** **Systematic reviews with meta-analyses (AD vs. other dementias): sensitivity and specificity values**.

CSF biomarker and Study	Number of studies included	Number of AD patients	Number of non-AD demented patients	Sensitivity (95% CI)	Specificity (95% CI)	LR+	LR−
**Aβ_42_**							
Bloudek et al. (2011)	12	ns	ns	73 (67–78)	67 (62–72)	2	0.4
**T-TAU**							
Bloudek et al. (2011)	19	ns	ns	78 (72–83)	75 (68–81)	3	0.3
Van Harten et al. (2011)	19	473	DLB: 208	73 (62–84)	90 (85–95)	7	0.3
	22	1330	FTLD: 464	74 (66–82)	74 (66–81)	3	0.4
	23	1048	VaD: 306	73 (60–86)	86 (80–90)	5	0.3
	7	175	CJD: 110	91 (86–96)	98 (97–100)	46	0.09
**p-TAU**							
Mitchell (2009)	18	1304	588	72 (63–80)	78 (72–83)	3	0.4
Bloudek et al. (2011)	20	ns	ns	79 (72–84)	80 (71–86)	4	0.3
Van Harten et al. (2011)	9	531	DLB: 210	74 (68–80)	83 (76–89)	4	0.3
	14	607	FTLD: 249	79 (67–90)	83 (76–90)	5	0.3
	13	333	VaD: 165	88 (72–92)	78 (68–88)	4	0.15
**COMBINATION OF Aβ_42_ AND T-TAU**							
Bloudek et al. (2011)	7	ns	ns	86 (79–91)	67 (53–79)	3	0.2

*AD, Alzheimer’s disease; CI, confidence interval; LR+, positive likelihood ratio; LR−, negative likelihood ratio; ns, non-specified; sensitivity and specificity values are expressed in percentages; DLB, dementia with Lewy bodies; FTLD, frontotemporal lobe degeneration; VaD, vascular dementia; CJD, Creutzfeldt–Jakob disease*.

**Table 4:** **Systematic reviews with meta-analyses (MCI-C vs. MCI-S): sensitivity and specificity values**.

CSF biomarker and study	Number of studies included	Number of MCI-C patients	Number of MCI-S patients	Sensitivity (95% CI)	Specificity (95% CI)	LR+	LR−
**Aβ_42_**							
Monge-Argilés et al. (2010)	6	128	216	67 (59–75)	71 (65–78)	2	0.5
**T-TAU**							
Monge-Argilés et al. (2010)	11	213	383	82 (76–86)	70 (65–75)	3	0.3
**p-TAU**							
Mitchell (2009)^a^	6	163	225	81 (69–91)	65 (50–80)	2	0.3
Monge-Argilés et al. (2010)	7	157	306	81 (75–87)	76 (70–81)	3	0.3
**COMBINATION OF Aβ_42_ AND T-TAU**							
Monge-Argilés et al. (2010)	3	101	164	81 (72–88)	87 (81–91)	6	0.2

MCI, mild cognitive impairment (MCI-C: MCI-converters, MCI-S: MCI-stables); CI, confidence interval; LR+, positive likelihood ratio; LR−, negative likelihood ratio; sensitivity and specificity values are expressed in percentages;

*^a^MCI patients who progress to dementia (non-necessarily AD)*.

#### 4.1.3 AD vs. healthy controls

##### 4.1.3.1 Aβ_42_

Decreased CSF Aβ_42_ concentrations to about 50% have frequently been reported in AD patients when compared to healthy controls. However, no reductions and even higher levels have also been reported. Sunderland et al. (2003) included 17 studies in their meta-analyses. A clear reduction in Aβ_42_ was found in 14 studies, while 2 studies were equivocal (Fukuyama et al., 2000; Csernansky et al., 2002), and another reported increased levels of Aβ_42_ in the AD group (Jensen et al., 1999). About the diagnostic utility of CSF Aβ_42_, Bloudek et al. (2011) reported a mean sensitivity of 80% (95% CI = 73–85%) and specificity of 82% (95% CI = 74–88%).

##### 4.1.3.2 T-tau

An increase by approximately 300% in the total concentration of tau in CSF has been found in many studies comparing AD patients vs. normal controls. In the meta-analysis performed by Sunderland et al. (2003), significant differences in CSF T-tau levels were obtained in all reviewed studies. About the diagnostic utility of T-tau, Bloudek et al. (2011) reported sensitivity of 82% (95% CI = 76–87%) and specificity of 90% (95% CI = 86–93%). As it can be seen in Table 2, T-tau has the highest LR+ (=8), indicating moderate increase in the likelihood of the disease.

##### 4.1.3.3 p-tau

p-tau concentration in CSF is also increased in AD when compared to healthy controls. Mean sensitivity values are around 80% [Mitchell (2009): 78%, with 95% CI = 71–84%; Bloudek et al. (2011): 80%, with 95% CI = 70–87%], while specificity is lightly higher [Mitchell (2009): 88%, with 95% CI = 84–91%; Bloudek et al. (2011): 83%, with 95% IC = 75–88%]. In addition, according to the meta-analyses performed by Mitchell (2009), PPV and NPV values were 90 and 73%, respectively. P-tau would facilitate 81.8 correct diagnoses for every 100 individuals tested. Different tau epitopes (p181, p199, and p231) had similar values, showing no significant differences between them.

##### 4.1.3.4 Combination of CSF core biomarkers

Only Bloudek et al. (2011) included the combination of several CSF core biomarkers on their meta-analyses. Results for the combination of Aβ_42_ and tau through 11 different studies gave a mean sensitivity of 89% (95% CI = 84–92%) and a mean specificity of 87% (95% CI = 83–90%). Moreover, as it is detailed in Table 2, the combination of Aβ_42_ and T-tau has the lowest LR− (=0.1), with moderate decrease in the likelihood of the disease.

#### 4.1.4 AD vs. other dementias

##### 4.1.4.1 Aβ_42_

The meta-analyses performed by Bloudek et al. (2011) showed that CSF Aβ_42_ distinguished AD patients from non-AD demented patients with a sensitivity of 73% (95% CI = 67–78%) and a specificity of 67% (95% CI = 62–72%). However, different forms of dementia were pooled together. We have not found further systematic reviews with meta-analyses comparing AD against other specific forms of dementia.

##### 4.1.4.2 T-tau

The meta-analyses performed by Bloudek et al. (2011) showed that CSF T-tau distinguished AD patients from non-AD demented patients with a sensitivity of 78% (95% CI = 72–83%) and a specificity of 75% (95% CI = 68–81%). In addition, Van Harten et al. (2011) published a detailed meta-analysis reporting sensitivity and specificity values for the differential diagnosis of AD against other specific entities as DLB, FTLD, VaD, and CJD. Regarding DLB, in spite of the considerably variability between studies, CSF T-tau levels are generally much lower in DLB than in AD. The meta-analyses performed by Van Harten et al. (2011) yielded a mean sensitivity of 73% (95% CI = 62–84%) and specificity of 90% (95% CI = 85–95%). CSF T-tau levels are also much lower in FTLD than AD. Since FTLD usually occurs before the age of 65, comparison with early-onset AD is relevant. When only patients with early-onset AD were analyzed, differences between FTLD and AD were even larger. In van Harten’s meta-analyses, sensitivity and specificity were both 74% (sensitivity: 95% CI = 66–82%; specificity: 95% CI = 66–81%). T-tau CSF concentrations in VaD patients are far lower than in AD patients. In the same study, Van Harten et al. (2011) reported a sensitivity of 73% (95% CI = 60–86%) and specificity of 86% (95% CI = 80–90%). Finally, numerous studies have shown that CJD is characterized by extremely high CSF T-tau values as compared with AD (at least 10-fold higher). Van Harten et al. (2011) obtained a sensitivity of 91% (95% CI = 86–96%) and specificity of 98% (95% CI = 97–100%). Table 3 shows likelihood ratios for the two meta-analyses. Results indicate that when comparing AD vs. CJD, T-tau offers an extremely high capacity to rule-in AD patients (LR+ = 46) and rule-out non-AD cases (LR− = 0.09). Moreover, T-tau turned out to be moderately appropriated to rule-in AD patients when compared to DLB (LR+ = 7) and VaD (LR+ = 5).

##### 4.1.4.3 p-tau

The diagnostic utility of CSF p-tau for AD against other dementias has been meta-analyzed by Bloudek et al. (2011); Mitchell (2009), and Van Harten et al. (2011). Both Mitchell and Bloudek presented sensitivity and specificity values for AD compared with a pooled group of different forms other non-AD dementias. Bloudek et al. (2011) obtained slightly higher sensitivity and specificity values (sensitivity: 79%, 95% CI = 72–84%; specificity: 80%, 95% CI = 71–86%). Mitchell (2009) reported a mean sensitivity of 72% (95% CI = 63–80%) and specificity of 78% (95% CI = 72–83%), with a PPV of 86% and NPV of 58%. Thus, p-tau would facilitate 73.7 correct diagnoses for every 100 individuals with dementia tested. An analysis about the specific p-tau epitopes showed that p181 appeared to be significantly less sensitive than either p199 or p231. In addition, p231 was significantly less specific than either p199 or p181. However, given the limited data for p199 and p231 these findings must be considered provisional. On the other hand, Van Harten et al. (2011) reported sensitivity and specificity values for the differential diagnosis of AD against specific forms of non-AD dementias. In relation to DLB, CSF p-tau levels were lower than in AD, with a sensitivity of 74% (95% CI = 68–80%) and specificity of 83% (95% CI = 76–89%). CSF p-tau levels in FTLD were also lower than in AD. Sensitivity was 79% (95% CI = 67–90%), and specificity 83% (95% CI = 76–90%). Regarding VaD, CSF p-tau values were also lower than in AD, with a sensitivity of 88% (95% CI = 72–92%), and specificity of 78% (95% CI = 68–88%). Patients with combined AD and VaD have elevated concentrations of T-tau and P-tau compared with AD patients. Finally, Van Harten et al. (2011) also presented some considerations for CJD related to AD. CSF p-tau alone has not been sufficiently investigated as diagnostic marker to differentiate both diagnostic categories. However, various studies indicate that CSF p-tau concentrations on CJD are relatively less increased compared with concentrations of T-tau. Moreover, two original studies combining T-tau and P-tau values showed very good diagnostic performance when comparing CJD and AD patients, with a sensitivity of 91–100% and specificity of 97–100% (Buerger et al., 2006; Matsui et al., 2010). Likelihood ratios presented in Table 3 show that p-tau is suitable to rule-in AD patients when compared to FTLD (LR+ = 5), and to rule-out non-AD cases when compared to VaD (LR− = 0.15).

##### 4.1.4.4 Combination of CSF core biomarkers

Bloudek et al. (2011) reported in their meta-analysis a mean sensitivity of 86% (95% CI = 79–91%) and specificity of 67% (95% CI = 53–79%) when combining CSF levels of Aβ_42_ and tau. This combination did not significantly increase the likelihood of AD vs. other dementias (LR+ = 3), but could be suitable to rule-out non-AD cases in the same context (LR− = 0.2).

#### 4.1.5 MCI-C vs. MCI-S

##### 4.1.5.1 Aβ_42_

At baseline, MCI-C have lower levels of CSF Aβ_42_ as compared to MCI-S, to controls, and even to those who have any additional decline yet not sufficient to reach the diagnostic threshold for dementia or AD (MCI-P) (Diniz et al., 2008; Schmand et al., 2010). Moreover, in the meta-analyses carried out by Diniz et al. (2008), CSF Aβ_42_ levels were similar for MCI-C and AD patients. Only one study demonstrated a significant reduction of CSF Aβ_42_ between baseline and follow-up assessments in MCI-C patients (Andreasen et al., 2003). Interestingly, another study showed that MCI patients with lower CSF Aβ_42_ values had a faster progression to AD (Herukka et al., 2007). In regards to the diagnostic utility of CSF Aβ_42_ to distinguish MCI-C from MCI-S, Monge-Argilés et al. (2010) reported a sensitivity of 67% (95% CI = 59–75%), and a specificity of 71% (95% CI = 65–78%).

##### 4.1.5.2 T-tau

MCI-C and MCI-P have higher CSF T-tau levels at baseline as compared to MCI-S patients and controls. In contrast, MCI-C and AD patients have similar CSF T-tau levels (Diniz et al., 2008; Schmand et al., 2010). Monge-Argilés et al. (2010), reported a sensitivity of 82% (95% CI = 76–86%), and a specificity of 70% (95% CI = 65–75%).

##### 4.1.5.3 p-tau

Cerebrospinal fluid p-tau levels in MCI-C patients are also higher at baseline as compared to MCI-S and controls (Diniz et al., 2008; Schmand et al., 2010). Regarding the diagnostic utility of CSF p-tau to distinguish MCI-C from MCI-S, Monge-Argilés et al. (2010) reported a sensitivity of 81% (95% CI = 75–87%) and specificity of 76% (95% CI = 70–81%). Moreover, Mitchell (2009) studied CSF p-tau ability to distinguish between MCI patients who progress to dementia (not necessarily AD), and MCI-S. Sensitivity was also 81% (95% CI = 69–91%). However, mean specificity fell down to 65% (95% CI = 50–80%). According to Mitchell’s analyses, p-tau would be expected to facilitate 71.9 correct diagnoses for every 100 individuals tested. The predicted PPV would be 63% and the NPV 83%, suggesting that p-tau might be best used to predict who would not progress rather than who might deteriorate.

##### 4.1.5.4 Combination of CSF core biomarkers

According to the meta-analysis performed by Diniz et al. (2008), in general, the association of two or three different CSF biomarkers yielded higher sensitivity and specificity values than each biomarker alone. Monge-Argilés et al. (2010) indicated a mean sensitivity of 81% (95% CI = 72–88%), and a specificity of 87% (95% CI = 81–91%). Moreover, likelihood ratios show that this combination also has the best capacity to rule-in MCI-C patients (LR+ = 6) and to rule-out non-MCI-C patients (LR− = 0.2).

### 4.2 Primary studies published after the new revised diagnostic criteria

The specific search for studies published between May 2011 and September 2013 resulted in 26 unique eligible references. Fourteen refer to the diagnostic ability of CSF core biomarkers to discriminate between AD and healthy controls (Bjerke et al., 2011; Baldeiras et al., 2012; Ewers et al., 2012; Mattsson et al., 2012; Mouton-Liger et al., 2012; Parnetti et al., 2012; Westman et al., 2012; Yang et al., 2012; Bombois et al., 2013; Guo et al., 2013; Lampert et al., 2013; Le Bastard et al., 2013; Molinuevo et al., 2013; Toledo et al., 2013). Eight studies include patients with AD vs. other dementias (Bjerke et al., 2011; Bibl et al., 2012; de Rino et al., 2012; Irwin et al., 2012; Toledo et al., 2012; Gabelle et al., 2013; Le Bastard et al., 2013; Muñoz-Ruiz et al., 2013). Twelve studies describe the ability of CSF core biomarkers to differentiate between MCI-C and MCI-S (Buchhave et al., 2012; Ewers et al., 2012; Mattsson et al., 2012; Parnetti et al., 2012; Vos et al., 2012, 2013; Westman et al., 2012; Yang et al., 2012; Gaser et al., 2013; Liu et al., 2013; Monge-Argilés et al., 2013; Toledo et al., 2013). Table 5 summarizes the main characteristics of these studies and their results. Sensitivity and specificity values of each individual primary study are presented in Tables 6–8. Sensitivity and specificity values were in line with those reported in systematic reviews with meta-analyses. Results show that lot of effort has been put in the combination of different biomarkers, managing to achieve the highest sensitivity and specificity values. Most of the recent studies do not only combine the CSF core biomarkers between them by calculating their ratios but also apply logistic regression models and advanced multivariate statistical methods. These models allow combining the CSF core biomarkers with other disease markers (Bjerke et al., 2011; Westman et al., 2012; Yang et al., 2012). Furthermore, several recent studies have analyzed the utility of indexes as the AD-CSF-index (Molinuevo et al., 2013) and the disease state index (DSI) (Muñoz-Ruiz et al., 2013), and some procedures as the Predict AD tool (Liu et al., 2013). DSI and PredictAD tool combine the CSF core biomarkers with demographic data, APOE, cognitive tests, and neuroimaging data.

**Table 5:** **Summary of primary studies published after the new revised diagnostic criteria: mean sensitivity and specificity values**.

	Number of comparisons included	Sensitivity		Specificity		LR+	LR−
		Mean (SD)	Min– max	Mean (SD)	Min– max	
**AD VS. HEALTHY CONTROLS**							
Aβ_42_	11	83 (9)	63–97	80 (8)	67–92	4	0.2
T-tau	12	78 (9)	61–91	82 (14)	53–97	4	0.3
p-tau	12	78 (10)	61–89	77 (18)	37–92	3	0.3
Combination	25	87 (6)	70–98	84 (9)	53–97	5	0.2
**AD VS. OTHER DEMENTIAS**							
Aβ_42_	5	85 (5)	82–95	61 (24)	22–80	2	0.2
T-tau	4	75 (14)	61–92	71 (22)	40–93	3	0.4
p-tau	4	80 (6)	77–88	78 (15)	56–88	4	0.3
Combination	19	86 (10)	67–100	78 (14)	36–97	4	0.2
**MCI-C VS. MCI-S**							
Aβ_42_	9	79 (14)	55–91	63 (20)	36–96	2	0.3
T-tau	9	76 (12)	60–88	58 (17)	39–88	2	0.4
p-tau	7	78 (9)	64–85	56 (18)	30–90	2	0.4
Combination	19	84 (10)	57–98	63 (19)	36–95	2	0.3

*AD, Alzheimer’s disease; MCI, mild cognitive impairment (MCI-C: MCI-converters, MCI-S: MCI-stables); SD, standard deviation; LR+, positive likelihood ratio; LR−, negative likelihood ratio; sensitivity and specificity values are expressed in percentages*.

**Table 6:** **Primary studies published after the new revised diagnostic criteria (AD vs. Healthy Controls): sensitivity and specificity values**.

CSF biomarker and Study	AD diagnostic criteria	Number of AD patients	Number of healthy controls	Sensitivity	Specificity	Specifications
**Aβ_42_**						
Baldeiras et al. (2012)	NINCDS-ADRDA	170	35^a^	79	73	
Bombois et al. (2013)	NINCDS-ADRDA	91	37^b^	77	79	
Guo et al. (2013)	NINCDS-ADRDA	69	92	78	74	
Le Bastard et al. (2013)	Neuropathological	51	95	94	88	ELISA
				88	92	LUMINEX
Mattsson et al. (2012)	NINCDS-ADRDA	529	304	85	82	Age ≤64
				85	82	Age 65–74
				85	73	Age ≥75
Molinuevo et al. (2013)	NINCDS-ADRDA	238	103	87	67	
Mouton-Liger et al. (2012)	NINCDS-ADRDA	45	35^c^	97	89	
Parnetti et al. (2012)	NINCDS-ADRDA	28	28	63	79	
**T-TAU**						
Baldeiras et al. (2012)	NINCDS-ADRDA	170	35^a^	78	82	
Bombois et al. (2013)	NINCDS-ADRDA	91	37^b^	76	95	
Ewers et al. (2012)	NINCDS-ADRDA	81	101	64	78	
Guo et al. (2013)	NINCDS-ADRDA	69	92	61	85	
Le Bastard et al. (2013)	Neuropathological	51	95	69	94	ELISA
				82	87	LUMINEX
Mattsson et al. (2012)	NINCDS-ADRDA	529	304	85	74	Age ≤64
				85	53	Age 65–74
				85	61	Age ≥75
Molinuevo et al. (2013)	NINCDS-ADRDA	238	103	82	83	
Mouton-Liger et al. (2012)	NINCDS-ADRDA	45	35^c^	91	97	
Parnetti et al. (2012)	NINCDS-ADRDA	28	28	82	93	
**p-TAU**						
Baldeiras et al. (2012)	NINCDS-ADRDA	170	35^a^	82	91	
Bombois et al. (2013)	NINCDS-ADRDA	91	37^b^	87	92	
Ewers et al. (2012)	NINCDS-ADRDA	81	101	67	79	
Guo et al. (2013)	NINCDS-ADRDA	69	92	61	86	
Le Bastard et al. (2013)	Neuropathological	51	95	77	78	ELISA
				69	91	LUMINEX
Mattsson et al. (2012)	NINCDS-ADRDA	529	304	85	67	Age ≤64
				85	46	Age 65–74
				85	37	Age ≥75
Molinuevo et al. (2013)	NINCDS-ADRDA	238	103	82	72	
Mouton-Liger et al. (2012)	NINCDS-ADRDA	45	35^c^	89	90	
Parnetti et al. (2012)	NINCDS-ADRDA	28	28	64	89	
**COMBINATION OF CSF CORE BIOMARKERS**						
Baldeiras et al. (2012)	NINCDS-ADRDA	170	35^a^	80	94	T-tau/Aβ_42_
				88	91	Aβ_42_/p-tau
Bjerke et al. (2011)	NINCDS-ADRDA	30	30	93	83	OPLS^d^
Bombois et al. (2013)	NINCDS-ADRDA	91	37^b^	88	89	T-tau/Aβ_42_
				89	92	Aβ_42_/T-tau
				84	97	Hulstaert’s index^e^
Guo et al. (2013)	NINCDS-ADRDA	69	92	78	79	LR^f^
Lampert et al. (2013)	NINCDS-ADRDA	62	74	92	66	T-tau/Aβ_42_ (all)
		37	36	95	53	T-tau/Aβ_42_ (FH+^g^)
		25	38	88	79	T-tau/Aβ_42_ (FH−^h^)
Le Bastard et al. (2013)	Neuropathological	51	95	98	91	LR^i^ (ELISA)
				94	87	LR^i^ (LUMINEX)
Mattsson et al. (2012)	NINCDS-ADRDA	529	304	85	95	Age ≤64 (LR^j^)
				85	83	Age 65–74 (LR^j^)
				85	80	Age ≥75 (LR^j^)
Molinuevo et al. (2013)	NINCDS-ADRDA	238	103	84	88	T-tau/Aβ_42_
				84	87	p-tau/Aβ_42_
				94	86	AD-CSF-index for T-tau^k^
				94	84	AD-CSF-index for p-tau^l^
				93	81	Hulstaert’s index^m^
Parnetti et al. (2012)	NINCDS-ADRDA	28	28	78	89	Aβ_42_/T-tau
				83	82	Aβ_42_/p-tau
Toledo et al. (2013)	NINCDS-ADRDA	92	110	70	82	T-tau/Aβ_42_
Westman et al. (2012)	NINCDS-ADRDA	96	111	84	79	OPLS^n^
Yang et al. (2012)	NINCDS-ADRDA			87	75	SVM^o^

*AD, Alzheimer’s disease*.

*^a^Neurological controls, most of them suffering from acute or chronic headaches with no subjective cognitive complaints*.

*^b^Controls consisted of individuals with psychiatric disorders or other neurological conditions such as headaches, dysarthria, chronic alcoholism, Parkinson’s disease, multiple sclerosis or drug addiction*.

*^c^Neurological controls*.

*^d^OPLS: Orthogonal projection to latent structures. The biomarkers contributing the most to the discrimination between AD and controls were in the following order: Aβ_42_, T-tau, NF-L (neurofilament light), MMP-10 (myelin basic protein), p-tau-181, H-FABP (heart fatty acid binding protein), and MBP (matrix metalloproteinases)*.

*^e^Hulstaert’s index: Aβ_42_/[p-tau(3.694 + 0.0105 T-tau)] (see Hulstaert et al., 1999)*.

*^f^LR: Logistic regression model including Aβ_42_, T-tau, and p-tau-181*.

*^g^FH+: Family history positive. First-degree relative who has developed late-onset AD*.

*^h^FH-: Family history negative*.

*^i^LR: Logistic regression model including Aβ_42_ and T-tau*.

*^j^LR: Logistic regression model including Aβ_42_/p-tau, and T-tau*.

*^k^AD-CSF-index for T-tau: [(Aβ_max_ – Aβ_42_)/(Aβ_max_ – Aβ_min_) + (T-tau – T-tau_min_)/(T-tau_max_ – T-tau_min_)], where Aβ_max_ and T-tau_max_ represent the 95^th^ percentile of the respective values, and Aβ_min_ and T-tau_min_ represent the 5^th^ percentile of the distribution values)*.

*^l^AD-CSF-index for p-tau: [(Aβ_max_ – Aβ_42_)/(Aβ_max_ - Aβ_min_) + (p-tau – p-tau_min_)/(p-tau_max_ – p-tau_min_)]*.

*^m^Hulstaert’s index: Aβ_42_/(240 + 1.18tau)*.

*^n^OPLS: The model included Aβ_42_, T-tau and p-tau*.

*^o^SVM: Linear support vector machine, including Aβ_42_, T-tau and p-tau*.

**Table 7:** **Primary studies published after the new revised diagnostic criteria (AD vs. other dementias): sensitivity and specificity values**.

CSF biomarker and Study	AD diagnostic criteria	Number of AD patients	Number of non-AD demented patients	Sensitivity	Specificity	Specifications
**Aβ_42_**						
Bibl et al. (2012)	NINCDS-ADRDA	22	17 (FTLD)	95	53	
de Rino et al. (2012)	NINCDS-ADRDA	72	42 (FTLD)	82	22	
Gabelle et al. (2013)	NINCDS-ADRDA	272	405 (AA, FTLD, LBD, ODD, OND, PSY)	82	71	
Le Bastard et al. (2013)	Neuropathological	51	15 (CJD, DLB, FTLD, VaD, PDD, SCA)	84	79	ELISA
				84	80	LUMINEX
**T-TAU**						
de Rino et al. (2012)	NINCDS-ADRDA	72	42 (FTLD)	61	73	
Gabelle et al. (2013)	NINCDS-ADRDA	272	405 (AA, FTLD, LBD, ODD, OND, PSY)	81	78	
Le Bastard et al. (2013)	Neuropathological	51	15 (CJD, DLB, FTLD, VaD, PDD, SCA)	65	93	ELISA
				92	40	LUMINEX
**p-TAU**						
de Rino et al. (2012)	NINCDS-ADRDA	72	42 (FTLD)	88	56	
Gabelle et al. (2013)	NINCDS-ADRDA	272	405 (AA, FTLD, LBD, ODD, OND, PSY)	77	88	
Le Bastard et al. (2013)	Neuropathological	51	15 (CJD, DLB, FTLD, VaD, PDD, SCA)	77	80	ELISA
				77	87	LUMINEX
**COMBINATION**						
Bibl et al. (2012)	NINCDS-ADRDA	22	17 (FTLD)	91	65	Aβ_42_/Aβ_40_
				82	82	Aβ_42_/Aβ_38_
Bjerke et al. (2011)	NINCDS-ADRDA	30	26 (MD or sVaD)	89	90	OPLS^b^
de Rino et al. (2012)	NINCDS-ADRDA	72	42 (FTLD)	84	61	T-tau/Aβ_42_
				80	68	p-tau/Aβ_42_
Gabelle et al. (2013)	NINCDS-ADRDA	272	405 (AA, FTLD, LBD, ODD, OND, PSY)	87	79	Aβ_42_/T-tau
				85	84	Aβ_42_/p-tau
				85	87	LR^c^
Irwin et al. (2012)	Neuropathological	11	10 (FTLD)	100	91	T-tau/Aβ_42_ (ELISA)
	Clinical diagnosis^a^	30	10 (FTLD)	90	97	T-tau/Aβ_42_ (LUMINEX)
		11	10 (FTLD)	100	36	T-tau/Aβ_42_ (ELISA)
		30	6 (FTLD)	67	87	T-tau/Aβ_42_ (LUMINEX)
Le Bastard et al. (2013)	Neuropathological	51	15 (CJD, DLB, FTLD, VaD, PDD, SCA)	88	80	LR^d^ (ELISA)
				90	73	LR^d^ (LUMINEX)
Muñoz-Ruiz et al. (2013)	DSM-IV	35	37 (FTLD)	70	71	DSI^e^
						
Toledo et al. (2012)	Neuropathological	71	29 (FTLD)	90	82	LR^f^ (ELISA)
				100	88	LR^g^ (LUMINEX)
						
	**NIA-AA**	71	29 (FTLD)	69	80	LR^h^ (ELISA)
				79	78	LR^h^ (LUMINEX)

*AD, Alzheimer’s disease; FTLD, frontotemporal lobe degeneration; AA, amyloid angiopathies; DLB, dementia with Lewy bodies; ODD, other neurodegenerative diseases; ND, other non-neurodegenerative diseases; PSY, psychiatric disorders; CJD, Creutzfeldt–Jakob disease; VaD, vascular dementia; PDD, Parkinson’s disease dementia; SCA, spinocerebellar ataxia; MD, mixed dementia; sVaD, subcortical vascular dementia*.

*^a^Clinical diagnosis: criteria applied is not specified*.

*^b^OPLS: Orthogonal projection to latent structures. The biomarkers contributing the most to the discrimination between AD and sVaD were in the following order: MBP (matrix metalloproteinases), TIMP-1 (tissue inhibitor of metalloproteinases), p-tau-181, NF-L (neurofilament light), T-tau, MMP-9 (myelin basic protein), Aβ_42_, and MMP-2 (myelin basic protein)*.

*^c^LR: logistic regression model including p-tau and Aβ_42_*.

*^d^LR: logistic regression model including Aβ_42_ and p-tau*.

*^e^DSI: disease state index, including APOE, Aβ_42_, T-tau, p-tau, MRI (hippocampal volume, tensor-based morphometry, and voxel-based morphometry), and MMSE*.

*^f^LR: logistic regression model including Aβ_42_ and T-tau*.

*^g^LR: logistic regression model including Aβ_42_ and p-tau*.

*^h^LR: logistic regression model including T-tau and p-tau*.

**Table 8:** **Primary studies published after the new revised diagnostic criteria (MCI-C vs. MCI-S): sensitivity and specificity values**.

CSF biomarker and Study	AD diagnostic criteria	Number of MCI-C patients	Number of MCI-S patients	Sensitivity	Specificity	Specifications
**Aβ_42_**						
Buchhave et al. (2012)	NINCDS-ADRDA	72	62^a^	90	76	
Gaser et al. (2013)	NINCDS-ADRDA	32	33	91	36	MCI (early progression)^d^
		66	33	89	36	MCI (all)^e^
Mattsson et al. (2012)	NINCDS-ADRDA	271	102^b^	85	77	Age ≤64
				85	56	Age 65–74
				85	60	Age ≥75
Parnetti et al. (2012)	NINCDS-ADRDA	32	58	56	96	
Vos et al. (2013)	NINCDS-ADRDA	130	216	75	58	Amnestic MCI
		61	131	55	71	Non-amnestic MCI
**T-TAU**						
Ewers et al. (2012)	NINCDS-ADRDA	58	72	61	59	
Gaser et al. (2013)	NINCDS-ADRDA	32	33	84	39	MCI (early progression)^d^
		66	33	88	39	MCI (all)^e^
Mattsson et al. (2012)		271	102^b^	85	65	Age ≤64
				85	49	Age 65–74
				85	46	Age ≥75
Parnetti et al. (2012)	NINCDS-ADRDA	32	58	62	88	
Vos et al. (2013)	NINCDS-ADRDA	130	216	74	61	Amnestic MCI
		61	131	60	78	Non-amnestic MCI
**p-TAU**						
Ewers et al. (2012)	NINCDS-ADRDA	58	72	64	59	
Gaser et al. (2013)	NINCDS-ADRDA	32	33	78	58	MCI (early progression)^d^
		66	33	68	58	MCI (all)^e^
Mattsson et al. (2012)		271	102^b^	85	55	Age ≤64
				85	44	Age 65–74
				85	30	Age ≥75
Parnetti et al. (2012)	NINCDS-ADRDA	32	58	81	90	
**COMBINATION**						
Buchhave et al. (2012)	NINCDS-ADRDA	72	62^a^	88	90	Aβ_42_/p-tau
				82	94	(Aβ_42_/p-tau) + T-tau
Gaser et al. (2013)	NINCDS-ADRDA	32	33	97	42	Aβ_42_/p-tau (MCI early prog.)^d^
		66	33	92	42	Aβ_42_/p-tau MCI (all)^e^
Liu et al. (2013)	**NIA-AA**^c^	158	233	90	36	Aβ_42_ or T-tau
				57	70	Aβ_42_ and T-tau
				73	71	PredictAD tool^f^
Mattsson et al. (2012)	NINCDS-ADRDA	271	102^b^	85	86	Age ≤64 (LR^g^)
				85	65	Age 65–74 (LR^g^)
				85	61	Age ≥75 (L^g^)
Monge-Argilés et al. (2013)	**NIA-AA**^c^	15	15	73	73	Aβ_42_, T-tau and p-tau-181
Parnetti et al. (2012)	NINCDS-ADRDA	32	58	94	65	Aβ_42_/T-tau
				81	95	Aβ_42_/p-tau
Toledo et al. (2013)	NINCDS-ADRDA	61	61	80	46	T-tau/Aβ_42_
Vos et al. (2012)	NINCDS-ADRDA	48	105	83	65	Aβ_42_/T-tau
Vos et al. (2013)	NINCDS-ADRDA	130	216	98	38	Aβ_42_/T-tau (amnestic MCI)
		61	131	90	54	Aβ_42_/T-tau (non-amnestic MCI)
Westman et al. (2012)	NINCDS-ADRDA	81	81	76	54	OPLS^h^
Yang et al. (2012)	NINCDS-ADRDA	25	86	80	44	SVMs^i^

*AD, Alzheimer’s disease; MCI, mild cognitive impairment; MCI-C, MCI-converters; MCI-S, MCI-stables*.

*^a^MCS-S pooled together with MCI patients that developed other types of dementia at follow-up, including vascular dementia (*n* = 14), dementia with Lewy bodies (*n* = 3), frontotemporal lobe degeneration (*n* = 1), Semantic Dementia (*n* = 2), and traumatic brain injury dementia (*n* = 1)*.

*^b^MCI-S pooled together with MCI patients that developed other types of dementia at follow-up, including vascular dementia (*n* = 28), dementia with Lewy bodies (*n* = 14), frontotemporal lobe degeneration (*n* = 7), and neurological diseases and dementia (*n* = 10)*.

*^c^MCI patients were classified according to the likelihood of AD conversion based on biomarker evidence of AD pathophysiological process (NIA-AA diagnostic criteria: Albert et al., 2011)*.

*^d^MCI (early progression): MCI patients that converted to AD within the first 12 months of follow-up*.

*^e^MCI (all): MCI early progression + MCI late progression (MCI patients that converted to AD after the first 12 months of follow-up)*.

*^f^PredictAD tool: including demographic data, APOE, MMSE, ADAS-Cog, neuropsychological battery, Aβ_42_, T-tau, and several features derived from MRI using FreeSurfer, manifold learning, hippocampal volume, and Tensor-Based Morphometry*.

*^g^LR: Logistic regression model including Aβ_42_/p-tau, and T-tau*.

*^h^OPLS: The model included Aβ_42_, T-tau and p-tau*.

*^i^SVM: Linear support vector machine, including Aβ_42_, T-tau and p-tau*.

It must be noticed that only three studies applied the new revised diagnostic criteria for AD or classified the MCI patients according to biomarker evidence of AD pathophysiology (Toledo et al., 2012; Liu et al., 2013; Monge-Argilés et al., 2013). In addition, although not reporting sensitivity and specificity values, we detected six further studies that also applied the new revised diagnostic criteria (Heister et al., 2011; Galluzzi et al., 2013; Knopman et al., 2013; Prestia et al., 2013; Roe et al., 2013). Galluzzi et al. (2013), Heister et al. (2011), Monge-Argilés et al. (2013) and Prestia et al. (2013) found that progression to AD was more frequent in MCI patients with increased biological severity based on biomarkers. Galluzzi et al. (2013), reported that 100% of MCI patients with the AD biomarker pattern developed AD, but 0% of the patients with normal biomarker pattern did so. Heister et al. (2011) and Monge-Argilés et al. (2013) mostly replicated these results. Moreover, Prestia et al. (2013) showed that conversion from MCI to AD is not only more frequent among individuals with biomarker positivity but also occurs earlier. Interestingly, two very recent studies have reported that individuals in the preclinical AD phase (cognitively normal but with biomarker positivity) have an increased rate of conversion to MCI (21%) compared to controls with a normal biomarker profile (7%) (Knopman et al., 2013), and also have a more rapid progression (Roe et al., 2013).

Finally, some authors are trying to improve the diagnostic performance of the CSF core biomarkers by controlling for different factors. For instance, difficulties in predicting MCI progression to AD could be influenced by the intrinsic heterogeneity of MCI. Recent studies show several aspects that directly affect the predictive power of the biomarkers, and should thus be taken into account when designing future studies and interpreting previous results. Vos et al. (2013) found that AD biomarkers might not be as sensitive in non-amnestic MCI as in amnestic MCI. Buchhave et al. (2012) found that baseline CSF Aβ_42_ levels were equally reduced in patients with MCI who converted to AD within 0–5 years (early converters) compared with those who converted between 5 and 10 years (late converters). However, CSF T-tau and p-tau levels were significantly higher in early converters. This might potentially affect aspects like biomarkers combination or prediction of early/late converters. Buchhave et al. (2012) showed that biomarkers combination resulted in a reduction in the negative predictive value because many patients with MCI who developed AD after 5–10 years had normal T-tau levels at baseline. Results reported by Gaser et al. (2013) show that CSF core biomarkers had generally better performance for early converters (<12 months) than for late converters (>12 months). Other studies show the influence of factors such as the age in biomarkers performance. Mattsson et al. (2012) found that although the diagnostic accuracies for AD decreased with age, the predictive values for a combination of biomarkers remained essentially stable. Finally, other authors have focused in factors as family history of AD. Lampert et al. (2013) showed that when comparing AD patients and healthy controls, T-tau/Aβ_42_ showed better sensitivity for individuals with family history of AD, but worse specificity compared to individuals without family history of AD.

## 5 Discussion

This meta-review includes seven studies identified in the literature as systematic reviews with meta-analysis on the topic of CSF core biomarkers for AD: Bloudek et al. (2011); Diniz et al. (2008); Mitchell (2009); Monge-Argilés et al. (2010); Schmand et al. (2010); Sunderland et al. (2003), and Van Harten et al. (2011). Moreover, it must be emphasized that, according to our systematic review, no systematic reviews with meta-analysis have been published after reviewed criteria for AD were published (May 2011). Therefore, we also carried out a specific search of primary studies published from May 2011 to the date of our search (September 2013). Twenty-six primary studies served as the focus for this synthesis. In total, the included systematic reviews with meta-analysis comprise 317 references, of which 130 are unique or non-repeated. Seventy-two references compare AD vs. healthy controls (Aβ_42_: 30, T-tau: 50; p-tau: 26), 78 studies analyze the discrimination between AD and other dementias (Aβ_42_: 11, T-tau: 60; p-tau: 41), and 23 articles compare MCI-C vs. MCI-S (Aβ_42_: 13, T-tau: 20; p-tau: 15). The diagnostic ability of CSF biomarkers to differentiate AD from healthy controls and other dementias are the two aspects that have received more attention in the literature. Regarding the specific biomarkers, a total of 39 non-repeated references focused on Aβ_42_, 103 on T-tau and 60 on p-tau. Noteworthy, Aβ_42_ is the less frequently studied biomarker, in spite of its core involvement in AD, whereas T-tau stands out as the most studied.

Regarding the diagnostic ability of the different CSF core biomarkers, this meta-review confirms that the combination provides the highest values of sensitivity and specificity. This is likely due to that they reflect two aspects of AD pathology, i.e., plaques (Aβ_42_), and neurodegeneration (tau). This combination seems to be useful for distinguishing between AD patients and healthy controls, as well as predicting which MCI patients will progress to dementia. If the situation would require the use of a single biomarker, T-tau has the highest values of sensitivity and specificity when comparing AD and healthy controls. However, no single biomarker at present is appropriate to differentiate MCI-C from MCI-S. Nevertheless, the only systematic review with meta-analysis in the literature concerning prediction of MCI was limited to three original studies (Monge-Argilés et al., 2010). Revision of primary studies published between 2011 and 2013 helps to clarify this issue. Sensitivity values are lower than reported by Monge-Argilés et al. (2010) and the specificity is clearly suboptimal. However, considering factors such as age, family history of AD and several aspects inherent to MCI heterogeneity could help to improve the predictive performance of CSF biomarkers. For instance, CSF core biomarkers are more effective in young MCI patients (<64 years) (Mattsson et al., 2012) amnestic MCI cases (Vos et al., 2013) and early converters (<12 months) (Gaser et al., 2013).

On the other hand, CSF core biomarkers seem to fail in distinguishing AD from other dementias, both when used as single biomarkers or in combination. The reason is that both CSF T-tau and Aβ_42_ levels are partially overlapped between AD and DLB, FTLD, and VaD (Buerger et al., 2007; Hampel et al., 2010; Van Harten et al., 2011). The combination of different CSF biomarkers provides the highest sensibility (86%), but is quite unspecific (67%) (Bloudek et al., 2011). An exception occurs in the case of CJD, where T-tau shows optimal performance in discriminating CJD from AD (Van Harten et al., 2011). In this meta-review, p-tau arose as the CSF biomarker with the best performance for differentiating AD from other dementias, although with sensitivity and specificity values around 75 and 80%, respectively (Mitchell, 2009; Bloudek et al., 2011; Van Harten et al., 2011). The explanation for this outperforming may be that p-tau is not a simple marker of axonal damage and neuronal degeneration, as T-tau, but it is more closely related to AD physiopathology and the formation of neurofibrillary tangles (Anoop et al., 2010; Holtzman, 2011). In addition, CSF p-tau concentrations seem to be more control-like and less AD-like in DLB, FTLD, and VaD (Van Harten et al., 2011). Interestingly, different p-tau isoforms might have differential pathophysiological roles in AD (Buerger et al., 2007; Engelborghs et al., 2007). There is some evidence indicating that P-tau_231_ may improve the differentiation between AD and FTLD (Buerger et al., 2002; Hampel et al., 2004), while p-tau_181_ may improve the differentiation between AD and DLB, and AD and VaD (Buerger et al., 2002; Hampel et al., 2004). P-tau_396–404_, and the ratio of p-tau_396–404_/T-tau has been shown in one study to differentiate AD from VaD (Hu et al., 2002). However, this promising results must been confirmed in future studies.

The analysis of likelihood ratios provides some valuable hints, supporting and complementing sensitivity and specificity figures reported in previous literature and discussed above. Briefly, T-tau is appropriated to rule-in AD patients when compared to healthy controls, DLB and VaD, and is conclusive when compared to CJD. Moreover, p-tau shows good capacity to rule-in AD cases vs. FTLD, and to rule-out non-AD patients when compared to VaD. Combination of CSF biomarkers is the best option to rule-out non-AD cases when compared to healthy controls and mixed groups of non-AD dementia. It is also the best option to rule-out non-MCI-C cases, as well as, to rule-in MCI-C patients.

Although the combination of CSF biomarkers provides the best diagnostic performance, only two systematic reviews with meta-analysis analyzed such issue (Monge-Argilés et al., 2010; Bloudek et al., 2011). Furthermore, together the two meta-analyses included only 14 original studies. In this meta-review we also analyze 26 further studies published after the new revised diagnostic criteria. Several findings deserve special attention. P-tau/Aβ_42_ ratio possesses higher sensitivity and specificity for differentiating AD from healthy controls and from other dementias, as compared to T-tau/Aβ_42_ ratio (Maddalena et al., 2003; Holtzman, 2011). For instance, p-tau/Aβ_42_ ratio seems promising in group separation between AD and VaD (Jong et al., 2006). The combination of p-tau/Aβ_42_ could also efficiently predict progression from MCI to AD with high efficiency (Hansson et al., 2006; Mattsson et al., 2009; Buchhave et al., 2012; Parnetti et al., 2012; Roe et al., 2013). Interestingly, increased tau/Aβ_42_ ratio in normal individuals has been associated with an increased risk of conversion from normal to MCI/very mild dementia in four recent studies (Fagan et al., 2007; Li et al., 2007; Craig-Schapiro et al., 2010; Roe et al., 2013). These and other studies support the utility of the CSF biomarkers to predict appearance of clinical symptoms in cognitively normal individuals that are at the preclinical phase of AD, or have cognitive complaints, or harbor some genetic risk (Skoog et al., 2003; Moonis et al., 2005; Fagan et al., 2007; Gustafson et al., 2007; Li et al., 2007; Stomrud et al., 2007; Ringman et al., 2008; Craig-Schapiro et al., 2010; Nettiksimmons et al., 2010; Fortea et al., 2011; Rami et al., 2011; Bateman et al., 2012; Holland et al., 2012; Desikan et al., 2013; Roe et al., 2013; Van Harten et al., 2013). In addition, the combination of Aβ_42_ and Aβ_40_ might be also useful in AD diagnosis and for the differential diagnosis vs. other dementias (Spies et al., 2010). Although several studies have focused on this ratio and reported interesting results (Vigo-Pelfrey et al., 1993; Mehta et al., 2000; Lewczuk et al., 2003; Wiltfang et al., 2003; Schoonenboom et al., 2005; Bentahir et al., 2006; Kumar-Singh et al., 2006; Hansson et al., 2007), this area remains controversial and deserves more research. Therefore, since these indexes appear to have the highest diagnostic efficiency, and since different combinations are possible, future work should pursue in this direction.

In summary, the diagnostic performance of CSF core biomarkers for AD is generally satisfactory, with sensitivity and specificity values above 80%. CSF core biomarkers are optimal for discriminating AD patients from healthy controls. This perhaps is an artificial contrast not representative of realistic clinical comparisons, but may have a useful application in research and clinical trials (Petersen and Trojanowski, 2009). The combination of CSF core biomarkers could also be suitable to predict which MCI patients will progress to dementia. Several recent studies support the utility of CSF core biomarkers for MCI prognosis (Vos et al., 2012; Choo et al., 2013; Galluzzi et al., 2013; Prestia et al., 2013). Single CSF core biomarkers provide unsatisfactory specificity values (50–81%) (Monge-Argilés et al., 2010). However, prediction of MCI-C by CSF biomarkers could be optimized using longer observation periods (>6 years) (Jong et al., 2006; Mattsson et al., 2009) and controlling several factors as age, MCI subtype and family history of AD. Related to this, is the fact that the predictive value and biomarkers’ utility strongly depend on the stage of the disease and time to conversion. Buschhave et al. (Buchhave et al., 2012) showed that Aβ_42_ performs better than Tau or structural MRI 5–10 years before conversion to AD, but T-tau and p-tau have better predictive power 0–5 years before conversion to AD. The highest performance of structural MRI is close to AD conversion. In general, predictive power of advanced MRI techniques in conversion from MCI to AD is greater than of CSF biomarkers (Brys et al., 2009; Vemuri et al., 2009; Landau et al., 2010; Walhovd et al., 2010; Cui et al., 2011; Davatzikos et al., 2011; Schmand et al., 2012; Westman et al., 2012; Gaser et al., 2013), although some studies also show comparable predictive power (Jack et al., 2010b; Yang et al., 2012; Liu et al., 2013; Vos et al., 2013), or even better performance of the CSF biomarkers, especially when MRI biomarkers consisted on clinical measures of hippocampal volume (Bouwman et al., 2007; Eckerström et al., 2010; Vos et al., 2012). Therefore it is necessary to move forward in the study of CSF biomarkers and different combinations. Studies should not only combine the CSF core biomarkers with each other but also with other biomarkers. Recent studies show an increase in the diagnostic efficiency of CSF core biomarkers when combined with neuroimaging biomarkers (Vos et al., 2012; Westman et al., 2012; Choo et al., 2013; Galluzzi et al., 2013; Prestia et al., 2013; Shaffer et al., 2013).

Several limitations obstruct the spread of CSF core biomarkers to the clinical routine (Henry et al., 2013; Sperling and Johnson, 2013; Zetterberg and Blennow, 2013). First, sensitivity and specificity of the “ideal” biomarker to detect AD should be at least 80% (The Ronald and Nancy Reagan Research Institute of the Alzheimer’s Association and the national Institute on Aging working Group, 1998). Higher levels are not easy to be achieved given that analyses are derived from clinically diagnosed AD cases in which the diagnostic accuracy already approximates 85% when validated by the standard pathologic diagnosis at autopsy (Mendez et al., 1992; Victoroff et al., 1995). A recent study showed that the use of clinical diagnosis instead of neuropathological diagnosis led to a 14–17% underestimation of the CSF biomarker accuracy (Toledo et al., 2012). With the new revised criteria the hope is to accomplish higher correspondence between clinical diagnosis and definitive AD postmortem confirmation. It is also necessary to test the CSF core biomarkers in pathologically confirmed AD patients. However, only a few studies have addressed this issue (Shaw et al., 2009; Brunnström et al., 2010; De Jager et al., 2010; Irwin et al., 2012; Toledo et al., 2012; Le Bastard et al., 2013). For this reason, we did not include a specific section in the current meta-review. Indeed, further original studies are mandatory before we can extract definitive conclusions regarding the diagnostic performance of CSF core biomarkers when compared to pathologically confirmed AD cases. Finally, since AD is a multifactorial neurodegenerative disorder both at clinical and neuropathological level, development of biomarkers with 100% efficiency in terms of sensitivity and specificity is difficult to achieve.

A second limitation is the variability between studies in the characteristics of the groups included and the diagnostic criteria used. This is true at different levels. Regarding healthy controls, in some occasions individuals with subjective memory complaints and neurological or psychiatric patients have been included as controls (Nägga et al., 2002; Buerger et al., 2003; Schoonenboom et al., 2004; Mitchell, 2009; Mouton-Liger et al., 2012; Bombois et al., 2013). Other studies have mixed healthy controls together with MCI-S patients (Diniz et al., 2008). It is even more alarming that quite many studies actually do not clearly specify what kind of participants are included as healthy controls. As lumbar puncture is not easily achieved in healthy volunteers, an amalgamate of non-demented patients is usually included instead. Regarding MCI, AD and other dementias, a relevant aspect is the lack of standardization in the clinical criteria used for diagnoses, especially for VaD. MCI is a heterogeneous condition (Petersen, 2004), having a large percentage of them an underlying diagnosis that is not AD (Fagan et al., 2007; Shaw et al., 2009). In AD studies, AD-like MCI is necessary to be guaranteed. Recently revised diagnostic criteria for MCI (Albert et al., 2011) can add great benefit to this regard. Other aspect that critically affects sensitivity and specificity values is the great heterogeneity in follow-up periods among MCI studies (Diniz et al., 2008). Studies with longer follow-up periods normally provide higher diagnostic efficiency (Jong et al., 2006; Mattsson et al., 2009). In relation to AD, most studies so far have used the NINCDS-ADRDA criteria, although a small percentage of studies have applied different criteria instead (Schmand et al., 2010). New proposed criteria must still be tested (McKhann et al., 2011). A critical issue is the possible circularity for the study of CSF biomarkers, given that now they are part of the diagnostic criteria. Regarding studies analyzing the comparison between AD and other dementias, an aspect that also affects the results and conclusions is that different forms of non-AD dementias are usually pooled together (Mitchell, 2009; Bloudek et al., 2011). Therefore, we highly recommend and encourage that future studies clearly specify groups’ characteristics, especially in regard to the control group, as well as the diagnostic criteria used for pathological groups. Also studies should specify whether sporadic or familial AD cases are included or in what proportion, in case they are combined. Likewise, age and sex should be accounted for as confounding factors.

A third limitation is the variability in methodological aspects of the technique in itself. Different organizations as the International Alzheimer’s Association (AA), the Alzheimer’s Biomarkers Standardization Initiative (ABSI), or The Penn Biomarker Core on Alzheimer’s Disease Neuroimaging Initiative (ADNI), are carrying out intense efforts to standardize the technical procedures. The AA has recently begun a program of quality control (QC) on CSF biomarkers for AD. Preliminary conclusions indicate that the standardization of laboratory procedures could contribute to reduce variability in the results and increase the utility of these biomarkers (Hansson et al., 2006; Fagan et al., 2011; Mattsson et al., 2011). Likewise, the ABSI has done an important contribution reviewing potential pre-analytical factors influencing the quantitative outcomes of AD biomarker assays and providing several recommendations [see Vanderstichele et al. (2012)].

In relation to the absence of a technical standardization is the variability in cut-off values to interpret CSF core biomarker levels. Differences between studies may reflect differences in laboratory methods, suggesting an inter-laboratory variation of results (Lewczuk et al., 2006). Recently, new methodologies have been introduced achieving less intra- and inter-assay variability as compared to standard methods such as ELISA (Innogenetics, Ghent, Belgium) (e.g., xMAP–Luminex) (Olsson et al., 2005). Standardized procedures are mandatory in order to obtain valid results. Due to inter-laboratory variability, at present, optimal cut-off values should be based on individual laboratory reference values rather than on values obtained from the literature. For this reason, proposing universal cut-offs values in this meta-review is difficult. Nevertheless, two options might temporarily solve this situation meanwhile strict standardizations are done. First, some authors suggest performing a systematic numeric normalization to account for this variability (Hansson et al., 2006). The exact variability this method introduces is unclear and deserves further specific review. Second, another potential solution is the novel proposal of a normalized index (the AD-CSF-index), which was recently validated to discriminate AD vs. controls in different European populations (Molinuevo et al., 2012, 2013). This index improves the diagnosis of AD by combining the normalized values of Aβ_42_ with T-tau or p-tau. It has shown higher sensitivity and specificity than the combination of direct values of the different CSF core biomarkers and avoids potential false positives associated with Aβ_42_ presence in the preclinical stage.

Finally, a fifth limitation concerns recruitment procedures. In high prevalence settings such as memory clinics where the prevalence of dementia is 30–50% (Feldman et al., 2003), reasonably high sensitivity and specificity values are expected. However, lower diagnostic performance is obtained in primary care, where the prevalence of dementia is approximately 15% (Ólafsdóttir et al., 2000). It is therefore necessary to specify patients source, as part of groups’ characteristics as we stated above. However, this information is not always provided in the studies. For instance, among the systematic reviews with meta-analysis included in this meta-review, only Mitchel (Mitchell, 2009) specified such information.

Cerebrospinal fluid core biomarkers remain quite promising. However, limitations discussed above must be urgently overcome. These CSF biomarkers tend to gain accuracy when assessed earlier in the disease process. We believe that this inherent characteristic should be promoted using them for the early diagnosis in preclinical stages of the disease and prediction from asymptomatic or MCI to AD. Studies with longer follow-up intervals in middle-age or elderly subjects who are normal at baseline are needed to test this potential. Regarding these kind of studies, research in normal subjects with increased risk for the development of AD is of great interest (Risacher and Saykin, 2013).

## 6 Conclusion and Perspective

This meta-review describes the state-of-the-art on CSF core biomarkers for AD in the context of new revised diagnostic criteria. Likewise, we offer a critical, integrated and systematic overview of the so far disperse information about the diagnostic efficiency and utility of CSF Aβ_42_, T-tau and p-tau, to distinguish AD from healthy controls, AD from other dementias, and to predict progression from MCI to AD. We have also thoroughly discussed main limitations in the field at the time being. A more detailed treatment of relevant issues such as performance of CSF core biomarkers in pathologically confirmed AD cases, heterogeneity of healthy and pathological groups, carelessness of confounding factors such as age and sex, and proposal of universal cut-offs values, are however far away from the aims of this meta-review. These are still open questions in the current literature and deserve much more specific revision.

Cerebrospinal fluid Aβ_42_, T-tau, and p-tau fulfill the criteria for diagnostically useful biomarkers in AD, and have been sufficiently validated in a large number of mono- and multi-center studies. They show potential usefulness for clinical practice due to their established ability to reduce misclassification rates when compared with the sole application of clinical/neuropsychological assessment (Mitchell et al., 2010). Moreover, in clinical trials, CSF core biomarkers can be useful to enrich the samples with pure AD cases, for patient stratification, as safety markers, and to detect and monitor the biochemical effects of drugs (Aluise et al., 2008; Hampel et al., 2008; Petersen and Trojanowski, 2009; Blennow et al., 2010).

However, despite promising results, CSF core biomarkers are not currently suitable for its wide implementation in the clinical routine as core elements for diagnostic criteria (Sperling and Johnson, 2013). Clinical diagnosis is still paramount and biomarkers are complimentary (Jack et al., 2011). This meta-review shows that CSF core biomarkers are optimal for discriminating AD patients from healthy controls. The combination of CSF biomarkers could be also suitable to predict which MCI patients will progress to dementia. However, CSF biomarkers fail at present to distinguish AD from other dementias. Recently revised criteria for AD include CSF core biomarkers together with neuroimaging biomarkers in the diagnostic algorithm (McKhann et al., 2011). Much additional work needs to be done to validate the application of biomarkers as they are proposed in new revised criteria. Nonetheless, CSF core biomarkers for AD show high potential value and leave room for improvement. In addition, other new candidate CSF biomarkers could potentially serve important functions in diagnostics and drug development if successfully validated in future studies (Rosén and Zetterberg, 2013). Upcoming investigations should also insist on plasma biomarkers, given that its use in the clinical routine is presumably easier. A more general use of CSF biomarkers in clinical practice will be of great importance. Suitable CSF biomarkers may help to diagnose AD at an early stage, which is of great importance when effective treatments for AD can be administered. Moreover, they may be used to monitor disease progression and target the right populations or used as an outcome measure for clinical trials.

## Conflict of Interest Statement

The authors declare that the research was conducted in the absence of any commercial or financial relationships that could be construed as a potential conflict of interest.

## Appendix

**Table A1:** **Search strategy for MEDLINE (OVID) database**.

1	*Alzheimer disease/cf, di (cerebrospinal fluid, diagnosis)
2	*Cognition disorders/cf, di, ge, ps (cerebrospinal fluid, diagnosis, genetics, pathology)
3	1 or 2
4	*Amyloid-beta-peptides/cf, du (cerebrospinal fluid, diagnostic use)
5	*Biological Markers/an, cf (analysis, cerebrospinal fluid)
6	*tau Proteins/cf (cerebrospinal fluid)
7	Phosphorylated tau.mp
8	Total-tau.mp
9	T-tau.mp
10	p-tau.mp
11	A beta-42.mp
12	*Cerebrospinal Fluid/an, di (analysis, diagnosis)
13	Cerebrospinal Fluid.mp.
14	CSF bioMarker.mp.
15	csf.mp.
16	Or/4-15
17	3 and 16
18	Limit 17 to “diagnosis (best balance of sensitivity and specificity)”
19	Limit 18 to humans

**Table A2:** **Search strategy for EMBASE (Elsevier) database**.

1	’alzheimer disease’/mj
2	’cognitive defect’/mj
3	1 OR 2
4	’amyloid-beta protein’/mj
5	’biological marker’/mj
6	’tau protein’/mj
7	’phosphorylated tau’:ab,ti
8	’total-tau’:ab,ti
9	’t-tau’:ab,ti
10	’p-tau’:ab,ti
11	’a beta-42’:ab,ti
12	’cerebrospinal fluid’/mj
13	’cerebrospinal fluid’:ab,ti
14	’csf biomarker’:ab,ti
15	csf:ab,ti
16	4 OR 5 OR 6 OR 7 OR 8 OR 9 OR 10 OR 11 OR 12 OR 13 OR 14 OR 15
17	3 AND 16
18	’sensitivity and specificity’/exp
19	sensitivity:ab,ti
20	specificity:ab,ti
21	((’pre test’ OR pretest) NEAR/8 probability):ab,ti
22	’post-test probability’:ab,ti
23	’predictive value?’:ab,ti
24	’likelihood ratio?’:ab,ti
25	’diagnostic accuracy’/mj
26	18 OR 19 OR 20 OR 21 OR 22 OR 23 OR 24 OR 25
27	17 AND 29

**Table A3:** **Strategies followed to reduce the risk of bias**.

**PUBLICATION BIAS AND REVIEWER SELECTION BIAS**	
1	Strict inclusion criteria for reviews
	Only systematic reviews where included, which attempts to collate all empirical evidence that fits pre-specified eligibility criteria. Systematic reviews use explicit and systematic methods that are selected to minimize bias
2	Manual query of relevant studies in systematic reviews
	Possible publication and reviewer selection bias in included systematic reviews was assessed and minimized by supplementing literature review with manual query of relevant studies within systematic reviews’ citations
3	Systematic review of primary studies
	Evidence was rigorously reviewed in order to minimize both publication and reviewer selection bias
4	Examination of missing results or data
	Both systematic reviews and primary studies were carefully examined for clues suggesting that there may be missing results or data.
**DATA AVAILABILITY BIAS**	
5	Assessments were completed independently by more than one reviewer
	Two reviewers (DF, LP) independently sought for detailed data in all identified studies. Peer review was done independently and in case of doubt and/or disagreements a third reviewer (PS) was consulted
**METHODOLOGICAL QUALITY**	
6	Oxman’s scale
	Methodological quality of included systematic reviews with meta-analyses was critically appraised with the Oxman’s scale. Assessment was performed in a blind manner by two reviewers (DF y LP), independently, and in case of doubt and/or disagreements, a third reviewer (PS) was consulted
7	PRISMA statement for reporting systematic reviews with meta-analyses
	Reporting transparence of the different systematic reviews was assessed with PRISMA checklist. Assessment was performed in a blind manner by two reviewers (DF y LP), independently, and in case of doubt and/or disagreements, a third reviewer (PS) was consulted

*DF: Daniel Ferreira; LP: Lilisbeth Perestelo-Pérez; PS: Pedro Serrano-Aguilar*.

**Table A4:** **Methodological quality of selected systematic reviews with meta-analyses according to Oxman’s Scale**.

Study	Total score (/10)	Clinical question (/2)	Criteria selection (/2)	Relevant studies covered (/2)	Validity (/2)	Combination of results (/2)
Bloudek et al. (2011)	8	2	2	2	0	2
Diniz et al. (2008)	8	2	2	2	1	1
Mitchell (2009)	8	2	1	2	2	1
Monge-Argilés et al. (2010)	7	2	2	1	1	1
Schmand et al. (2010)	7	2	2	1	0	2
Sunderland et al. (2003)	8	2	2	2	1	1
Van Harten et al. (2011)	7	2	2	2	0	1

**Table A5:** **PRISMA checklist of items for reporting systematic reviews with meta-analyses**.

Study	Title	Abstract	Introduction		Methods												Results							Discussion			Funding
Items	1	2	3	4	5	6	7	8	9	10	11	12	13	14	15	16	17	18	19	20	21	22	23	24	25	26	27
Bloudek et al. (2011)	✓		✓	✓^a^			✓	✓					✓	✓		✓	✓			✓	✓		✓	✓		✓	✓
Diniz et al. (2008)	✓		✓	✓^a^		✓	✓	✓	✓	✓	✓		✓				✓	✓		✓	✓			✓	✓	✓	✓
Mitchell (2009)	✓		✓	✓^a^	✓	✓	✓	✓	✓	✓	✓	✓		✓		✓	✓	✓		✓	✓		✓	✓	✓	✓	
Monge-Argilés et al. (2010)	✓		✓	✓		✓	✓	✓	✓				✓	✓			✓	✓		✓	✓			✓	✓	✓	
Schmand et al. (2010)	✓		✓	✓		✓	✓	✓	✓	✓	✓	✓	✓	✓	✓	✓	✓	✓	✓	✓	✓	✓	✓	✓	✓	✓	✓
Sunderland et al. (2003)			✓	✓^a^		✓	✓	✓	✓	✓			✓	✓			✓	✓		✓	✓			✓	✓	✓	✓
Van Harten et al. (2011)	✓		✓	✓		✓	✓	✓	✓				✓	✓				✓		✓	✓		✓	✓	✓	✓	

✓ – PRISMA item fulfilled;

*^a^objectives are explicitly stated but PICOS are not specified (PICOS, participants, interventions, comparisons, outcomes, and study design); PRISMA Items: (1) Title; (2) Structured summary; (3) Rationale; (4) Objectives; (5) Protocol and registration; (6) Eligibility criteria; (7) Information sources; (8) Search; (9) Study selection; (10) Data collection process; (11) Data items; (12) Risk of bias in individual studies; (13) Summary measures; (14) Synthesis of results; (15) Risk of bias across studies; (16) Additional analyses; (17) Study selection; (18) Study characteristics; (19) Risk of bias within studies; (20) Results of individual studies; (21) Synthesis of results; (22) Risk of bias across studies; (23) Additional analysis; (24) Summary of evidence; (25) Limitations; (26) Conclusions; (27) Funding*.
